# The Aneugenicity of Ketone Bodies in Colon Epithelial Cells Is Mediated by Microtubule Hyperacetylation and Is Blocked by Resveratrol

**DOI:** 10.3390/ijms22179397

**Published:** 2021-08-30

**Authors:** Haruka Sudo, Akira Kubo

**Affiliations:** Faculty of Health Science, Tokoha University, 1-30, Mizuochi-cho, Aoi-ku, Shizuoka-shi, Shizuoka 420-0831, Japan; ak@kuboakira.com

**Keywords:** colon cancer, aneuploidy, microtubule severing, diabetes

## Abstract

Diabetes mellitus (DM) is considered to be associated with an increased risk of colorectal cancer. Recent studies have also revealed that tubulin hyperacetylation is caused by a diabetic status and we have reported previously that, under microtubule hyperacetylation, a microtubule severing protein, katanin-like (KL) 1, is upregulated and contributes to tumorigenesis. To further explore this phenomenon, we tested the effects of the ketone bodies, acetoacetate and β-hydroxybutyrate, in colon and fibroblast cells. Both induced microtubule hyperacetylation that responded differently to a histone deacetylase 3 knockdown. These two ketone bodies also generated intracellular reactive oxygen species (ROS) and hyperacetylation was commonly inhibited by ROS inhibitors. In a human fibroblast-based microtubule sensitivity test, only the KL1 human katanin family member showed activation by both ketone bodies. In primary cultured colon epithelial cells, these ketone bodies reduced the tau protein level and induced KL1- and α-tubulin acetyltransferase 1 (ATAT1)-dependent micronucleation. Resveratrol, known for its tumor preventive and tubulin deacetylation effects, inhibited this micronucleation. Our current data thus suggest that the microtubule hyperacetylation induced by ketone bodies may be a causal factor linking DM to colorectal carcinogenesis and may also represent an adverse effect of them that needs to be controlled if they are used as therapeutics.

## 1. Introduction

Diabetes mellitus (DM) has been associated with increased risk of multiple cancer types, including colorectal cancer (CRC) [1,2]. Common risk factors such as obesity, physical inactivity, and smoking are likely to contribute to this increased CRC risk in DM patients. This CRC association with DM has been reported to be maintained when these factors are adjusted [3], indicating that diabetes itself includes tumor promoting mechanisms in its pathology. Although the biological mechanisms that may link DM to cancer are thought to include hyperglycemia, hyperinsulinemia, insulin-like growth factors, oxidative stress, and subclinical inflammation, they remain to be completely understood.

Ketone bodies have been well-established to be representative pathogenic substances in DM. They are small, lipid-derived molecules that serve as a circulating energy source for tissues in times of fasting or exercise. Ketone bodies include acetoacetate (AA), β-hydroxybutyrate (BHB), and acetone, and ketogenesis mainly occurs in the mitochondria of liver cells, which normally produce ketone bodies as the result of fatty acid breakdown when the blood glucose levels are low. Ketogenesis also occurs in kidney cells, brain astrocytes [4], and colon epithelial cells [5]. The levels of ketone bodies in the blood increases to 1–2 mM while fasting and to even higher concentrations during prolonged periods of fasting (6–8 mM) and in diabetic ketoacidosis (>25 mM). A ketogenic diet has been known to have therapeutic effects against epilepsy for many years [6]. It is also thought that ketone bodies in low concentrations have beneficial human health effects in multiple organs including anti-inflammatory properties [7] and renal protective effects against ischemia-reperfusion injury [8]. Early studies have also shown, however, that ketosis is associated with accelerated aging and increased ketone body levels in the body have been found to cause age-related metabolic disease [8]. Further to this, a recent report has indicated that the local synthesis of ketone bodies by 3-hydroxy-3-methylglutaryl-CoA synthetase 2 (HMGCS2) maintains intestinal stem cell homeostasis via the activation of Notch signaling [5].

In terms of an association with tumorigenesis, although the ketogenic diet has been evaluated for cancer prevention and treatment purposes with the hope of attenuating tumor growth via calorie restriction, there have been studies suggesting tumor promoting effects of ketosis. A chemical induction of DM in rats with streptozotocin has been reported to be sufficient to enhance tumor growth [9]. Similarly, acute fasting in rodent animal models has been reported to be sufficient to increase tumor growth [10]. Furthermore, a recent report in human breast cancers has indicated that mammary adipocyte-derived ketone bodies contribute to the malignant growth of breast tumors via an induction of epigenetic effects [11]. Ketone bodies are also thought to fuel breast cancer growth through a “reverse Warburg Effect” [12] and contribute to BRAF V600E-expressing human melanoma and hairy cell leukemia development [13]. Thus, it remains to be clarified whether and how ketone bodies contribute to carcinogenesis.

The studies of both human patients and an animal model of DM have shown that enhanced tissue tubulin acetylation is a characteristic of this disorder [14,15]. Previous research, including from our laboratory, has also demonstrated that microtubule hyperacetylation may have tumor promotive effects [16,17,18]. We have reported that microtubule hyperacetylation induces aneuploidy via enhanced katanin-like1 microtubule severing [18]. We therefore searched for causal substances in DM pathology that enhance microtubule acetylation and found that ketone bodies did have the effects, remarkably, in cells derived from colon epithelium.

## 2. Results

### 2.1. Ketone Bodies Enhance Microtubule Acetylation Both in Cancerous and Non-Cancerous Cells

**Figure 1:**
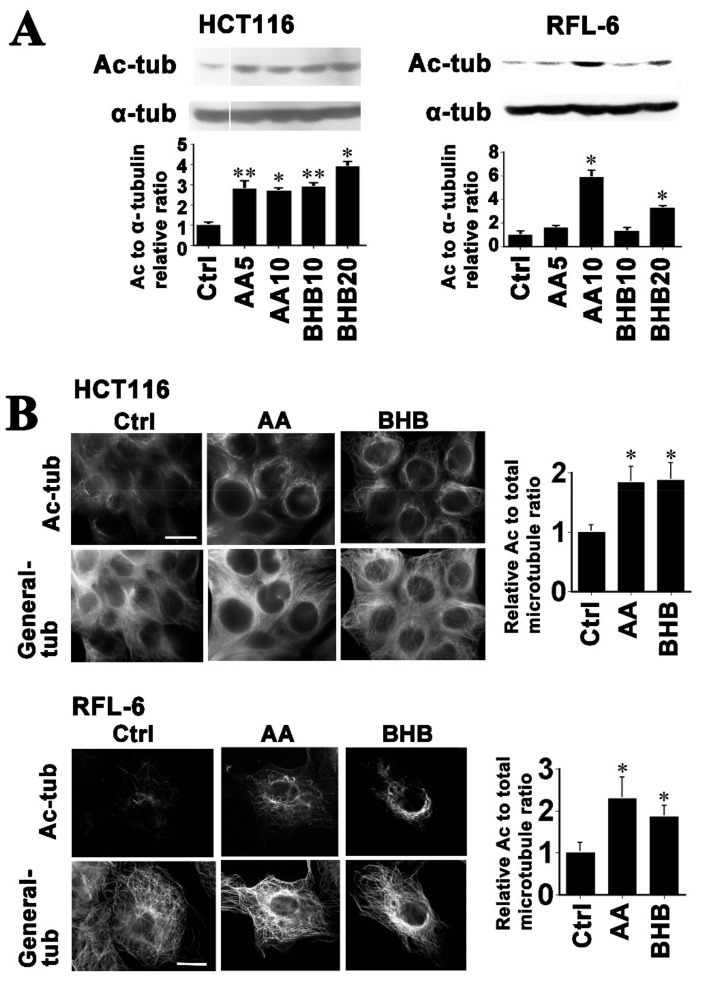
Ketone bodies have microtubule acetylation enhancement effects in RFL-6 and HCT116 cells. (**A**) HCT116 and RFL-6 cells were treated for three days with 5 or 10 mM AA, and 10 or 20 mM BHB, and whole cell lysates were then subjected to immunoblotting with the indicated primary antibodies (anti-acetylated α-tubulin: Ac-tub and anti-α-tubulin: α-tub). The relative Ac and α-tubulin ratios to the controls (Ctrl) by quantification are indicated in the graphs below respective blot images. Significant increases were detected in both cell types. (**B**) Cells were treated with 10 mM AA or, 20 mM BHB, cultured for three days, fixed, and stained for general- and acetylated-tubulin. In the immunofluorescence images, the upper panels indicate acetylated-tubulin (Ac-tub), the lower corresponding panels show general-tubulin (General-tub) staining. A perinuclear enhancement of microtubule acetylation was prominent following the ketone body treatments in both cell types. Scale bar, 10 µm. The graphs show the quantified relative acetylated-tubulin/total-tubulin ratios to the control. Significant increases were detected in the ketone body-treated cells in both cell types. The asterisks and double asterisks indicate significant differences compared with the controls (Student’s *t*-test, * p < 0.01 and ** p < 0.05, respectively).

To test the effects of exposure to the BHB and lithium salt of AA on cellular microtubules, we used the previously established HCT116 human colon cancer cell line [17] and RFL-6 rat fibroblast line, which is non-transformed cells [19]. Prior studies have reported ketone body concentrations as high as 25 mM in uncontrolled diabetes and that physiologically AA and BHB are found in either 1:2 or 1:3 ratios [20]. We thus treated cells with AA and BHB at 0, 5, and 10 mM, and 0, 10, and 20 mM, respectively, for three days. Western blot analysis (Figure 1A) revealed significant increases in the acetylated-tubulin/α-tubulin ratios. The relative ratios to the controls in HCT116 cells were 2.8 ± 0.4 (Student’s *t*-test, *p* < 0.05), 2.7 ± 0.2 (*p* < 0.01), 2.9 ± 0.2 (*p* < 0.05), and 3.9 ± 0.3 (*p* < 0.01), for AA at 5 and 10 mM, and BHB at 10 and 20 mM, respectively. In RFL-6 cells, these ratios were 1.6 ± 0.1, 5.9 ± 0.6 (*p* < 0.01), 1.3 ± 0.1, and 3.3 ± 0.2 (*p* < 0.01), for AA at 5 and 10 mM, and BHB at 10 and 20 mM, respectively (Figure 1A). These responses to ketone body treatments were evident at one and two days after treatment.

Supporting the immunoblotting data, increases were also observed in microtubule acetylation in the ketone body-treated cells, as assessed by immunofluorescence (Figure 1B). In both cell types we detected a prominent enhancement of microtubule acetylation in the perinuclear regions. A previous study has reported that a high-glucose treatment increases the cellular microtubule acetylation levels [21] and we had predicted high glucose as one of the causal factors for tubulin hyperacetylation in DM in our previous report [18]. However, we were unable in our present analyses to detect this effect to any significant degree in either HCT116 and RFL-6 cells (Figure S1A). We also tested lithium chloride treatments of at 5 and 10 mM in HCT116 cells and found no effects on microtubule acetylation (Figure S1B), indicating that the acetoacetate anion is the active component. We further evaluated the combination of 5 mM AA and 15 mM BHB (AA:BHB = 1:3), but detected no additive effects compared with AA or BHB alone (Figure S1C). Regarding tubulin detyrosination [22] and polyglutamylation [23,24], we detected significant 1.35 ± 0.1 (*p* < 0.01) and 1.38 ± 0.1 (*p* < 0.05)-fold increases in the detyrosinated-tubulin/α-tubulin ratio in AA at 5 and 10 mM treatment, respectively, and a significant 1.6 ± 0.1 (*p* < 0.01)-fold increase in the polyglutamylated-tubulin/α-tubulin ratio following 10 mM AA treatment of HCT116 cells. Notably however, the BHB treatment produced no enhancement of detyrosination or polyglutamylation (Figure S1D).

These data suggest that both ketone bodies have microtubule acetylation enhancement effects, either in transformed or non-transformed cells.

### 2.2. Mechanisms of Microtubule Hyperacetylation Induced by AA and BHB

**Figure 2:**
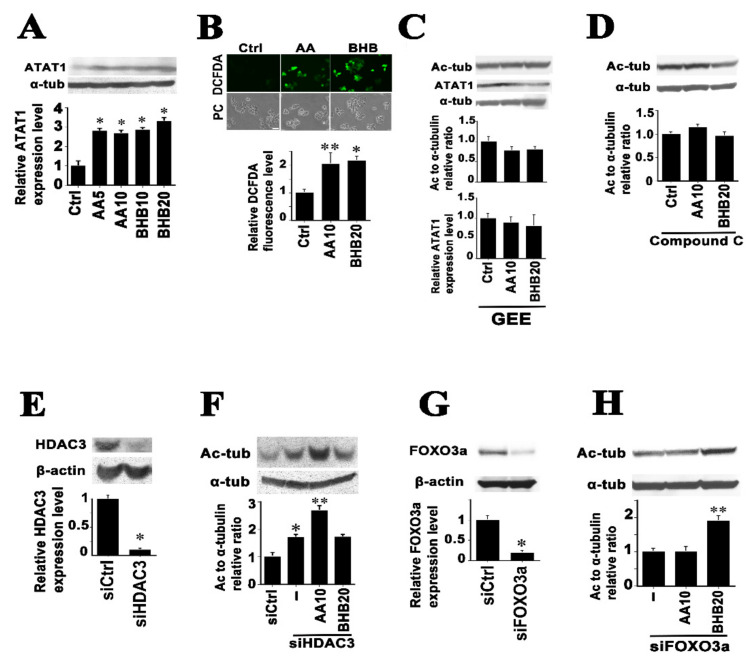
Characterization of the signal molecules for ketone body-induced microtubule hyperacetylation in HCT116 cells. (**A**) HCT116 cells were treated with the indicated concentrations of AA and BHB for three days, and whole cell lysates were then subjected to immunoblotting with the indicated primary antibodies (anti-ATAT1: ATAT1). The relative ATAT1 expression ratios to the controls are indicated in the bar graphs below the panels. Significant increases were detected following both AA and BHB treatments. (**B**) Cells were treated with 10 mM AA or 20 mM BHB for two days and then subjected to DCFDA staining to detect ROS. Significant elevations in ROS generation were detected following both ketone body treatments compared with the controls (upper images; DCFDA, lower images; phase contrast images of the corresponding upper images (PC)). Scale bar, 100 µm. The quantification of the DCFDA signals is indicated in the graph. (**C**) In the presence of 1 mM GEE, the HCT116 cells were treated with AA and BHB for three days. Western blot analysis revealed no significant increase in either the tubulin acetylation ratios or the ATAT1 protein levels compared with the controls. The graphs show the quantification results. (**D**) In the presence of 10 μM Compound C the HCT116 cells were treated with AA and BHB. No significant increase was detected in the tubulin acetylation ratios compared with the controls. (**E**) Cells were transfected with siRNAs against HDAC3. An approximately 90% reduction in the level of HDAC3 protein was achieved. (**F**) Under the HDAC3 knockdown, the effects of ketone bodies were examined by western blot. Compared with the control siRNA, an anti-HDAC3 siRNA transfection alone produced a significant increase in the tubulin acetylation level (asterisk in the graph). Compared with an anti-HDAC3 siRNA transfection alone, a significant increase in the tubulin acetylation ratio was detected following AA treatment (double asterisk in the graph) but not in the BHB-exposed cells. (**G**) Cells were transfected with siRNAs against FOXO3a. An approximately 80% reduction in the level of FOXO3a protein was achieved. (**H**) Under the FOXO3a knockdown, the effects of ketone bodies were examined. Compared with an anti-FOXO3a siRNA transfection alone, a significant increase in the tubulin acetylation ratio was detected following the BHB treatment (double asterisk in the graph) but not in the AA-exposed cells. The asterisks and double asterisks indicate significant differences (Student’s *t*-test, * p < 0.01 and ** p < 0.05, respectively).

To further elucidate the mechanisms by which the AA and BHB ketone bodies increase microtubule acetylation, we first examined whether the TCA cycle intermediates generated through ketone body oxidation in mitochondria are required to induce microtubule hyperacetylation. We tested the effects of the TCA cycle entry inhibitor aminooxyacetate (AOA) [7] for that purpose. Ketone body-induced hyperacetylation was observed as early as 2 h after treatment. AOA did not show significant inhibition on enhancement of microtubule acetylation (Figure S2A). This suggests that the ketone body molecules themselves may work as signaling factors. We next studied the expression levels of enzymes associated with α-tubulin lysine 40 acetylation (α-tubulin acetyltransferase 1 (ATAT1) [25,26]) or deacetylation (histone deacetylase 6 (HDAC6) [27], sirtuin 2 (SIRT2) [28], and HDAC3 [29]). We tested the endogenous protein expression levels of these enzymes in the lysates of HCT116 cells treated with AA or BHB. The enzyme that showed a significant change was ATAT1 (Figure 2A). Compared with the controls, the relative ATAT1 expression levels were 2.8 ± 0.1 (*p* < 0.01) and 2.7 ± 0.2 (*p* < 0.01), and 2.9 ± 0.1 (*p* < 0.01) and 3.3 ± 0.2 (*p* < 0.01), for AA at 5 and 10 mM, and BHB at 10 and 20 mM, respectively. The transcript levels of α-tubulin acetyltransferase 1 gene were examined by RT-PCR but showed no change (Figure S2B). It has been reported that both AA and BHB treatments cause intracellular reactive oxygen species (ROS) generation [20,30] and ROS have been shown to activate ATAT1 [26]. Hence, we next addressed whether there was ROS production in response to ketone body treatments by staining cells with the ROS probe DCFDA [20,31]. Under microscopy, we detected significantly increased green fluorescence in ketone body-treated cells (Figure 2B). We next examined the effects of the ROS inhibitors glutathione-ethyl-ester (GEE) [32] and *N*-acetyl-l-cysteine (NAC) [26]. Both agents showed inhibitory effects against microtubule hyperacetylation and the ATAT1 protein increases induced by ketone bodies (Figure 2C and Figure S2C). It has been reported that ROS activate AMPK [33], which leads to the activation of ATAT1 [26]. We therefore tested the effects of the AMPK inhibitor Compound C [34] and confirmed its inhibition effects also against microtubule hyperacetylation (Figure 2D). Consistently, we observed significant increases in AMPKα Thr172 phosphorylation, which is required for this kinase activation [33] in ketone body-treated cells (Figure S2D). These data suggest that a ROS-AMPK-ATAT1 axis operates in relation to AA- and BHB-induced microtubule hyperacetylation, which has similarities to previously reported mechanisms [26].

Ketone bodies have been reported to be endogenous HDAC inhibitors [35]. In particular, BHB is considered to be inhibitory against class I and class IIa HDACs [35,36]. Moreover, HDAC3, which belongs to the class I HDACs, has been shown to have indirect tubulin deacetylase activity [29,37,38,39]. Based on these prior findings, we next tested the involvement of HDAC3 in ketone body-mediated microtubule hyperacetylation. A more than 90% reduction of endogenous HDAC3 protein knockdown was attained by anti-HDAC3 siRNA transient transfection in HCT116 cells (Figure 2E). Transfection of siHDAC3 alone resulted in a 1.7-fold significant increase in microtubule acetylation compared with the control (1.7 ± 0.2) (Figure 2E). Interestingly, AA treated cells still showed a significant increase (1.6 ± 0.1) (*p* < 0.01) compared with the siHDAC3 alone, whereas BHB treated cells did not (1.0 ± 0.1), under a HDAC3 knockdown (Figure 2F). The association of BHB-induced microtubule acetylation with HDAC3 was further confirmed using an HDAC3 specific inhibitor RGFP966 [38] (Figure S2E). Ketone bodies have been demonstrated to induce forkhead transcriptional factor O3a (FOXO3a) [35]. A more than 80% reduction of FOXO3a was achieved by anti FOXO3a siRNA transfection (Figure 2G). Under this FOXO3a knockdown, cells under BHB treatment, but not AA exposure, showed a significant increase in microtubule acetylation. Different sensitivities to HDAC3 inhibitors and a FOXO3a knockdown appeared between AA and BHB, together with different responses in other posttranslational tubulin modifications, suggesting that the potentiation of microtubule acetylation is a biologically meaningful and common event in cells exposed to ketone rich environments.

### 2.3. Both AA and BHB Enhance Microtubule Severing by KL1

We have reported previously that microtubule acetylation increases the cellular microtubule sensitivity to katanin family microtubule severing proteins, rat katanin, and human KL1 [18,40]. In this regard, to clarify whether ketone bodies potentiate the microtubule severing activity of katanin family proteins through enhanced microtubule acetylation, we performed a fibroblast-based microtubule sensitivity test as described previously [18,19,40,41]. Because of our interest in human disease, we employed human lung fibroblast IMR90-SV cells [42] for this analysis, which show a relatively flat morphology. Moreover, because species differences in the function of microtubule-associated proteins have been reported [43], we tested the human versions of katanin (FKat), KL1 (FKL1), and KL2 (FKL2) that had all been N-terminally-tagged with a flag peptide.

**Figure 3:**
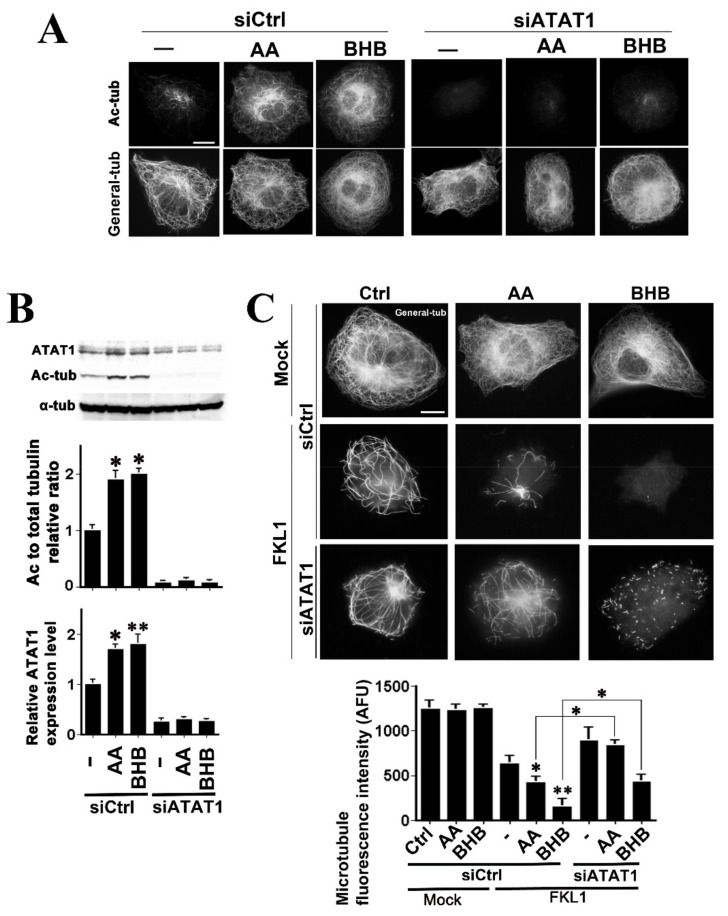
Effects of ketone body-induced microtubule acetylation on KL1-mediated microtubule severing in IMR90-SV cells. (**A**) Effects of ketone body treatment on cellular microtubule acetylation in IMR90-SV cells. Cells were transfected with an siRNA against ATAT1, treated with 10 mM AA or 20 mM BHB, cultured, and then stained with anti-tubulin and anti-acetylated-tubulin antibodies. In the immunofluorescence images, the upper panels indicate acetylated tubulin (Ac-tub), and the lower panels denote the corresponding general tubulin (General-tub) images. There was no difference between the untransfected cells and the control siRNA transfected cells. Compared with the control cells (siCtrl), both AA- and BHB-treated cells showed significant increase in microtubule acetylation. Under the ATAT1 knockdown, only a marginal acetylated-tubulin signal was detectable, regardless of the treatment by different ketone bodies. The quantification data are described in the results section. Scale bar, 10 µm. (**B**) Under the same set of conditions as in (**A**), immunoblotting was performed using anti-ATAT1, anti-acetylated-tubulin, and anti-α-tubulin antibodies. Quantification data are shown in the graphs below the panels. Significant increases in microtubule acetylation were detectable in the AA- and BHB-treated cells compared with the controls. The anti-ATAT1 siRNA transfection reduced the Ac to α-tubulin ratios to very low levels regardless of the presence of ketone bodies (upper graph). Anti-ATAT1 blots indicated significant increases in the response to AA or BHB treatments in the control cells. Under siATAT1 transfection, 70–80% reductions in ATAT1 levels were detected in both the untreated and ketone body-treated cells (lower graph). (**C**) Representative images of the microtubule sensitivity test results. Flag-KL1 (FKL1)-overexpressing cells showed a significant microtubule polymer mass reduction compared with the control mock-transfected cells (Mock). There was no difference in the microtubule reduction following a further control siRNA transfection. The AA treatment enhanced the microtubule reduction significantly (AA + FKL1 + siCtrl) compared with the untreated-FKL1 expressing cells (Ctrl + FKL1 + siCtrl). The BHB treatment caused even stronger enhancement (BHB + FKL1 + siCtrl). In the siATAT1-transfected cells, these enhancements were significantly inhibited (AA or BHB + FKL1 + siATAT1). The graphs indicate the quantification results. AFU, arbitrary fluorescence unit. Scale bar, 10 µm. The asterisks and double asterisks denote significant differences (Student’s *t*-test, * p < 0.01 and ** p < 0.05, respectively).

Whole cell increases were observed in tubulin acetylation in the ketone body-treated cells, as assessed by immunofluorescence. Peaks were observed in the perinuclear region, as seen in RFL-6 and HCT116 cells. To manipulate the microtubule acetylation levels, we transiently transfected an siRNA against ATAT1. The effects of ketone bodies on microtubule acetylation were largely canceled out by this knockdown. The control siRNA-transfected cells showed the same responses to ketone body treatments as those of the untransfected cells (Figure 3A). Quantitatively, compared with the controls, the relative acetylated- to total-microtubule ratios were 3.8 ± 0.2 (*p* < 0.01), and 3.6 ± 0.1 (*p* < 0.01) for 10 mM AA- and 20 mM BHB-treated cells, respectively, and 0.4 ± 0.1, 0.5 ± 0.1, and 0.7 ± 0.1, for untreated-, AA-, and BHB-treated cells under siATAT1 transfection, respectively. Western blot analysis confirmed these increases (Figure 3B). The relative ratios of acetylated-tubulin/α-tubulin to the controls were 1.9 ± 0.2 and 2.0 ± 0.1 for AA and BHB-treatments, and these ratios leveled to 0.1 for the same set of treatments under anti-ATAT1 siRNA transfection. Consistent with these data in the HCT116 cells (Figure 2A), we observed significant increases in the ATAT1 protein levels in response to ketone body treatments (relative ATAT1 levels to controls; 1.7 ± 0.1 and 1.8 ± 0.2 for AA- and BHB-treated cells, respectively; Figure 3B, lower graph). These elevations in ATAT1 expression were effectively repressed by ATAT1 siRNA transfection (0.2 ± 0.1, 0.3 ± 0.1, and 0.3 ± 0.1, for untreated-, AA- and BHB-treated cells under siATAT1 transfection, respectively), supporting the observed effects upon tubulin acetylation.

The siATAT1 transfections neither generated changes in the organization nor polymer mass of the microtubules. Compared with the mock-transfected control cells, treatments with 10 mM AA or 20 mM BHB caused no changes in the microtubule organization or concentration (Figure 3C, upper panels). Control siRNA transfections did not affect the sensitivities of the cells to exogenous microtubule severing proteins. The FKL1-expressing cells showed a significant 50% reduction in the microtubule levels. This downregulation was significantly enhanced by AA exposure (66% reduction). A stronger enhancement was observed, however, in the BHB-treated cells (87% reduction). In the BHB + KL1 experiment, we frequently observed cells devoid of microtubules (Figure 3C). These effects by ketone body treatments were significantly inhibited by siATAT1 transfection (Figure 3C, graph). FKat-expressing cells showed a significant (40%) reduction in the microtubule levels (Figure S3). This downregulation was significantly enhanced by AA (59% reduction) while there was no enhancement but in fact a repression in the BHB-treated cells. The enhancement by AA treatment was significantly inhibited by siATAT1 transfection (Figure S3, graph). FKL2 did not show significant microtubule severing activity as reported previously [44,45].

These data are concordant with our hypothesis that ketone bodies increase the microtubule severing activities of katanin family proteins through microtubule hyperacetylation. The observed inhibitory effects of BHB on FKat were unexpected however. Human katanin has been shown to be under strict control [46] and BHB has been known to have epigenetic effects [8,35]. Katanin selective inhibitor molecules might be induced.

### 2.4. Microtubule Hyperacetylation-Mediated Aneuploidization Is Induced by Ketone Bodies in Cells of Colon Epithelial Origin

Microtubule hyperacetylation has been shown to have carcinogenesis promotive effects [16,18] and ATAT1 transcript levels have been reported to be increased in human colon cancer tissues [17]. Hence, we first addressed whether there is an enhancement of microtubule acetylation in human colon cancer tissue lysates (Figure S4A). Out of eight evaluable patients represented on the commercial test strips that we tested, two showed increases in the relative ratios of acetylated- to α-tubulin in their tumors compared with the corresponding neighboring normal tissue (25%) and no patient showed a reduction. The relative ratios of the tumor to control signals were 2.4 ± 0.2, and 2.0 ± 0.2, for cases T7-018, and T7-044, respectively (mean signal intensity ± S.D., *n* = 3). These data support our hypothesis.

HCT116 cells have been demonstrated to be chromosomally stable [47] and we therefore tested the effects of ketone bodies on micronucleation, an indicator of genome instability. First, we checked the spindle microtubule acetylation status. Compared with the controls, 10 mM AA- and 20 mM BHB-treated cells showed significant increases in spindle microtubule acetylation (Figure S4B). Next, we detected abnormal mitosis spindle morphology [18,19] in both AA- and BHB-treated cells (Figure S4Ba; 2.5 ± 0.3%, 4.4 ± 0.1%, and 10.2 ± 2.1% for the control, AA-, and BHB-treated cells, respectively) while no change was observed in the mitotic index of the cells (1.6 ± 0.2%, 1.8 ± 0.5%, and 1.6 ± 0.6%, for control, AA-, and BHB-treated cells, respectively). Consistently, we detected a significant increase in micronucleation in the BHB-treated cells whereas AA-treated cells showed a tendency only towards an increase (Figure S4B). From the aspect of the tau protein status, the effects of BHB were not contrary to our hypothesis because tau is known to be phosphorylated in HCT116 cells [48].

**Figure 4:**
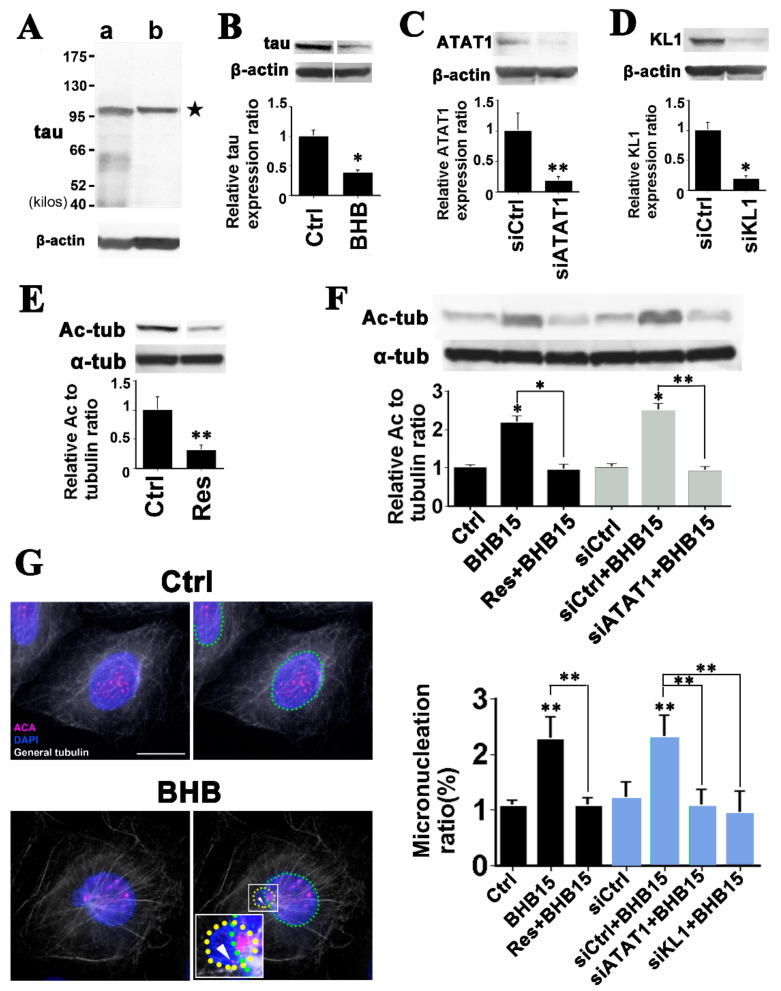
BHB induces whole chromosome micronucleation via upregulated microtubule acetylation in HCECs. (**A**) Tau protein expression in HCECs. Whole cell lysates of HCT116 cells (**a**) and HCECs (**b**) were subjected to immunoblotting with anti-tau antibody. HCT116 cells were used as a positive control and expressed various tau species including Big tau of near 95 kDa in size. HCECs showed only Big tau expression. The star denotes Big tau which has been shown to be an inhibitor of KL1 [19]. (**B**) Treatment of HCECs with 15 mM BHB for three days reduced the tau levels significantly, by around 70% (graph). (**C**) Effects of siATAT1 in HCECs. A more than 80% reduction was achieved. (**D**) Effects of the KL1 knockdown on HCECs. A more than 80% reduction was achieved. (**E**) Effects of resveratrol (Res) on microtubule acetylation in HCECs. HCECs were treated with 75 μM resveratrol, cultured for three days, and analyzed by immunoblotting. Compared with the controls, an approximately 70% reduction in the Ac to tubulin rate was evident in the resveratrol-treated cells. (**F**) Effects of resveratrol and siATAT1 on BHB-induced microtubule hyperacetylation. In the first experiments, HCECs were treated with 15 mM BHB, further treated with 75 μM resveratrol, and cultured for three days. Significant increases in tubulin acetylation were detected in response to BHB, which were inhibited by resveratrol (graph; black bars). In the second set of experiments, cells were transfected with anti-ATAT1 siRNAs for one day and then treated with 15 mM BHB, cultured for three days. Control cells showed significant increases in microtubule acetylation in response to BHB treatments (siCtrl + BHB15), whereas this enhancement was significantly suppressed by siATAT1 (siATAT1 + BHB15; gray bars in the graph). (**G**) ACA-positive micronucleation induced by BHB in HCECs. In the former experiments we detected a significant elevation in micronucleation (the right and left images are of the same cells but primary nuclei are encircled with a dotted green line and micronuclei with a dotted yellow line. In the control image, punctate ACA signals could be detected inside the primary nucleus indicating the positivity of ACA staining. There were no micronuclei evident in these controls. An ACA-positive micronucleus in addition to the primary nucleus was visible in the BHB image. The white arrowhead in the enlarged square denotes an ACA signal inside the micronucleus) in response to 15 mM BHB treatment for three days. In more than half of the micronuclei, we detected ACA signals in the BHB treated cells while only low levels (5%) were detectable in the control micronuclei (Ctrl). The increase in the micronucleation was suppressed by further treatment with resveratrol (Res + BHB15) (graph; black bars). In the latter experiments we transfected cells with anti-ATAT1 or anti-KL1 siRNAs for one day and then treated them with 15 mM BHB for three days. Compared with the controls, BHB treatment significantly enhanced the level of micronucleation, which was inhibited by siATAT1 (siATAT1 + BHB15) or siKL1 (siKL1 + BHB15) (graph; light blue bars). In the images, white indicates general tubulin, blue denotes DAPI, and magenta highlights the ACA signals. In the micronucleation counts under the microscope, more than 400 cells were counted under each of the conditions (*n* = 3). Scale bar, 10 µm. The asterisks and double asterisks indicate significant differences (Student’s *t*-test, * p < 0.01 and ** p < 0.05, respectively).

Since we observed significant effects of BHB upon HCT116 in micronucleation, we further tested this ketone body on primary cultured normal human colon epithelial cells (HCEC) that have been used in CRC studies [49]. Previously, we reported that the effects of microtubule hyperacetylation are masked if tau is intact [18,50]. It has been reported that the risk of developing cancer is significantly higher in families affected by genetic tauopathies [51] and MAPT is frequently methylated, with hypermethylation associated with a poorer prognosis, in CRC patients [52]. We therefore examined the expression of tau in HCECs and detected a subspecies, which is referred to HMW tau or Big Tau (Figure 4A(b)) [53]. This finding is consistent with those of previous reports [51,52,54]. Interestingly, the tau protein levels significantly decreased in response to 15 mM BHB treatment (Figure 4B). Compared with the controls, the relative tau protein expression level was 0.3 ± 0.1 (*p* < 0.01) in the BHB-treated HCECs.

We also transiently transfected HCECs with siRNAs against ATAT1 and KL1. Significantly, more than 80% reductions in both ATAT1 and KL1 proteins were achieved (Figure 4C,D). A natural polyphenol resveratrol (3, 4′, and 5-trihydroxystilbene), which is known for its anti-cancer effects [55,56], was included in our experiments. Resveratrol causes microtubule deacetylation by activating SIRT2, as described previously [57,58,59]. Consistently, we observed a significant near 70% reduction in the relative acetylated- to α-tubulin ratio when HCECs were treated with 75 μM resveratrol (Figure 4E). We next examined the effects of resveratrol and siATAT1 under BHB treatment conditions. Exposure of the cells to BHB at 15 mM significantly increased microtubule acetylation, which was efficiently repressed by further resveratrol addition (compared with the control, relative acetylated- to α-tubulin ratios were 2.2 ± 0.2 (*p* < 0.01) and 1.0 ± 0.2 for BHB alone and BHB + resveratrol, respectively; Figure 4F). We also observed an effective inhibition of BHB-induced microtubule hyperacetylation by ATAT1 knockdown (compared with controls, relative acetylated- to α-tubulin ratios were 2.5 ± 0.2 (*p* < 0.01) and 0.9 ± 0.1 (*p* < 0.05; vs. BHB) for BHB alone and BHB + siATAT1, respectively; Figure 4F).

Under these same experimental conditions, we further performed immunofluorescence analyses of the HCECs with nuclear and anti-centromere antigen (CREST; ACA) staining (Figure 4G, Ctrl images). BHB treatment generated a significant increase in whole chromosomes containing micronuclei (ACA-positive micronuclei) which is known an indicator of aneuploidization [18,19,60,61] (Figure 4G, BHB images). About 60% of the micronuclei were ACA-positive in BHB-treated cells, while few showed ACA positivity in the controls, suggesting a mitotic origin of BHB-induced micronuclei. Under a control siRNA transfection, we observed similar BHB-induced ACA-positive micronucleation, which was efficiently suppressed by the transfection of siRNAs against ATAT1 (1.1 ± 0.3%, 2.3 ± 0.4% (*p* < 0.01), and 1.1 ± 0.3% (*p* < 0.05; vs. BHB) for control, BHB, and BHB + siATAT1, respectively), supporting the microtubule acetylation-dependence of the micronucleation (Figure 4G, graph). Furthermore, the micronucleation was significantly inhibited by anti-KL1 siRNA transfection (0.9 ± 0.1%; *p* < 0.05; vs. BHB) supporting the causal role of KL1 (Figure 4G). Provocatively, resveratrol treatment efficiently suppressed the micronucleation (1.1 ± 0.2%; *p* < 0.05 vs. BHB; Figure 4G). The micronucleation was increased to 3.6% in BHB-treated p53 knockdown cells while no increase was detected in p53 knockdown alone cells (1.1%), which is consistent with the micronucleus clearance effects of p53 (*n* = 1) [60].

These results suggest that exposure to BHB generates aneuploidy, a tumor cell trait, in normal human colon epithelial cells.

## 3. Discussion

We have here evaluated the relationships between ketone bodies and carcinogenesis. Our data show that at a more than 10 mM concentration, both the AA and BHB ketone bodies commonly induce cellular microtubule hyperacetylation, whether the cells were transformed or not, and that this effect causes chromosome aberrations in colon epithelial cells. This may indicate that although a lower than 10 mM dose of a ketone body has beneficial effects for human health [8], which might include the effects on tumors of neuroectodermal origin, excessive levels of these compounds, even without showing severe toxic effects, seem to have a tumor promoting effect and may be a link between the increased risk of colon cancer in DM patients.

The mechanisms through which ketone bodies elicit microtubule hyperacetylation may involve stress response signal cascades driven by ROS [26]. BHB seems also to exhibit the activity as an endogenous inhibitor of HDAC3 in this role [35,36]. Although BHB has been known to act through the cell surface receptor GPR109 as a natural ligand [62], and GPR41 as an inhibitor of short-chain fatty acids [63], these receptors are likely to be dispensable for microtubule hyperacetylation because HCT116 cells do not express GPR109 [64] and no short-chain fatty acids were added to the culture medium in our current experiments.

The functional role of perinuclear region-centered microtubule hyperacetylation is an important question to emerge from our current study and prior reports. For example, NAD^+^ reduction causes similar perinuclear microtubule acetylation enhancement in stimulated macrophages [65]. The mitochondria are transported along with the acetylated microtubules to the endoplasmic reticulum (ER), upon which the NLRP3 adaptor protein ASC localized in the mitochondrion is capable of binding to other components of the NLRP3 inflammasome which is located in the ER, resulting in activation of the inflammasome. However, ketone bodies have a net inhibitory effect against NLRP3 inflammasome activation [7]. Given that ketone bodies are generally synthesized during stressful circumstances, we speculate that the close proximity of mitochondria can provide an efficient supply of ATP to the nucleus so that a nuclear anti-stress program can function well. In this scenario, the acetylated microtubules might function as a scaffold for the mitochondria. During prolonged stress, the perinuclear microtubule acetylation may be gradually decreased, as seen in the late phase of fasting [66]. Alternatively, if stress is relieved, the scaffold might be recognized and reorganized by katanin family severing proteins.

Adenomatous polyposis coli (*APC*) is known to be frequently mutated in human colon cancer. The APC^Min/+^ mouse is an established animal model of human adenomatous polyposis and these mice spontaneously develop multiple polyps in the small intestine [67]. BHB has been shown to be important for the mouse intestinal stem cell maintenance [5]. Deep in the intestinal crypt, intestinal stem cells express HMGCS2 and synthesize BHB endogenously for this purpose. In addition, the liver or other non-intestinal sources of exogenous ketone bodies increase the stem cell numbers and thereby upregulate regenerative activities after injury. This is because intestinal crypts have the ability to uptake systemically circulating ketone bodies [5]. Butyrate, a product of gut microbes, has been shown to promote colon carcinogenesis as an energy source [67] and has also been thought to increase ketone body production through HMGCS2 expression [68]. These insights suggest that the human colon tissue is under complex governance with respect to the ketone body environment and that the perturbation of this by DM might lead to carcinogenesis. Our current finding of an increased whole chromosome micronucleation in BHB-treated HCECs and the inhibitory effects of resveratrol on this process support this notion. Moreover, the anti-colon-carcinogenesis effects of resveratrol have been reported [55]. Colon tumor cells with an altered genome are known to be recognized by tumor-infiltrating lymphocytes [69]. The established inhibitory effects of ketone bodies against the NLRP3 inflammasome [7] may disrupt this anti-tumor mechanism however because this inflammasome has been suggested to influence tumor immunity by mediating tumor-infiltrating lymphocytes [70].

It is known that newborns have relatively high physiological blood concentrations of ketone bodies, which is advantageous for nervous system development as they help to build axon myelination [71]. Since microtubule severing proteins are associated with neurite outgrowth [41,72,73,74] and KL1 has been shown to affect neuronal morphology [75], ketone bodies may also have such developmental effects. In this regard, it is noteworthy that BHB has been shown to promote neurite outgrowth in cultured neurons [76].

Finally, although it produced milder effects compared with BHB, AA is an enhancer for both katanin and KL1. The concentration of BHB is generally considered higher than that of AA as mentioned earlier. The AA:BHB ratio of blood ketone bodies is determined by multiple factors including the redox state in liver mitochondria and some AA dominant pathological conditions have been described [77]. We predict that such conditions might also be tumor-prone due to the dual activation of the microtubule severing proteins.

## 4. Materials and Methods

### 4.1. Reagents and Antibodies

(R)-3-Hydroxybutyric acid (AdipoGen Life Sciences, SanDiego, CA, USA) was purchased and dissolved in phosphate-buffered saline, as described previously [8] (as BHB). Lithium acetoacetate (Sigma, St. Louis, MO, USA) was purchased and used as described previously (as AA) [78,79]. Lithium chloride anhydrous (Sigma) was also obtained commercially as was dorsomorphin (hydrochloride) (Cayman Chemical, Ann Arbor, MI, USA) (as Compound C). Glutathione ethyl seter, 2′, 7′-Dichlorofluorescein diacetate (DCFDA), and RGFP966, were also purchased from Cayman Chemical. Resveratrol was purchased from Combi-Blocks (San Diego, CA, USA). N-Acetyl-L-cysteine and (aminooxy) acetic acid were obtained from Fujifilm Wako Pure Chemical Corporation (Osaka, Japan). DAPI (4,6-diamidino-2-phenylindole dihydrochloride hydrate) was obtained from Sigma. D-glucose was purchased from Hayashi Pure Chemical IND. LTD. (Osaka, Japan).

Mouse monoclonal antibody anti-polyglutamylated tubulin, mAb (GT335) was purchased from Enzo Life Sciences (Ann Arbor, MI, USA). Commercially sourced mouse monoclonal anti-α-tubulin (DM1A, NeoMarkers, Fremont, CA, USA), Cy3-conjugated-β-tubulin (Sigma) (used for general tubulin staining), anti-SIRT2 (A-5), anti-HDAC3 (A-3), anti-β-actin (AC-15) (Santa Cruz Biotechnology, Inc., Dallas, TX, USA) and anti-acetylated-α-tubulin (clone 6-11B-1) (Sigma) were used in the experiments. Mouse monoclonal phospho-independent anti-Tau antibody (T1029) was obtained from USBiological (Swampscott, MA, USA). A rabbit polyclonal antibody against DDDDK-tag was used as the anti-Flag antibody (Medical and Biological Laboratories Co., Ltd., Nagoya, Japan). Rabbit polyclonal Anti-Tubulin Antibody, Detyrosinated (Millipore, Billerica, MA, USA) was also used. Rabbit polyclonal antibody against KATNAL1 (KL1; HPA046205) was obtained from Sigma and rabbit polyclonal anti-ATAT1 was sourced from Epigentek (Farmingdale, NY, USA) for use in ATAT1 detection. The detected major bands were isoform 3 (NCBI Reference Sequence: NP_001177653.1) in HCT116 cells and isoform 1 (NCBI Reference Sequence: NP_001026892.1) in both IMR90-SV cells and HCECs. Rabbit monoclonal anti-p53 antibody (clone 7F5, Cell Signaling Technology, Danvers, MA, USA) and anti-FOXO3A (ABclonal, Tokyo, Japan) were used as was a human anti-centromere antibody (Antibodies Inc., Davis, CA, USA) (as ACA). Rabbit anti-phospho-AMPKα (Thr-172) and anti-AMPKα1/2 were from Cell Signaling Technology.

### 4.2. Expression Constructs

Human FKat, FKL1, and FKL2 expression vectors were purchased from GenScript (Piscataway, NJ, USA): KATNA1_OHu05824C_pcDNA3.1 + N-DYK, KATNAL1_OHu10589C_pcDNA3.1 + N-DYK, and KATNAL2_OHu103129C_pcDNA3.1 + N-DYK, for FKat, FKL1, and FKL2, respectively.

### 4.3. Cell Culture, Transfection and Drug Treatments

#### 4.3.1. Fibroblasts

Rat RFL-6 fibroblasts were purchased from Health Protection Agency (Porton Down Salisbury, UK), and were cultured as described previously [18,19]. Human IMR90-SV fibroblasts were provided by RIKEN BRC (RIKEN BRC, Ibaraki, Japan) through the National BioResource Project of the MEXT/AMED, Japan, and cultured under the same conditions as the RFL-6 fibroblasts, i.e., in low-glucose D-MEM (D6046; Sigma) supplemented with 20% fetal bovine serum. For immunoblotting analysis, approximately 2.0 × 10^5^ and 1.5 × 10^5^ cells/well were seeded onto six-well plates for RFL-6 and IMR90-SV cells, respectively. For immunofluorescence studies, approximately 1.0 × 10^5^ RFL-6 or IMR90-SV cells/dish were plated onto 3.5 cm glass-bottom dishes. In the microtubule sensitivity test IMR90-SV cells were plated one day before siRNA transfection, transfected with 4 μg of each plasmid using Nucleofector II (Lonza, Basel, Switzerland) with program X-005 from the manufacturer. The cells were plated on 3.5 cm glass-bottom dish (Matsunami, Osaka, Japan) as described previously [40]. The transfection efficiency was about 30–40%. Transfection of IMR90-SVs with anti-ATAT1 siRNAs was performed using RNAi Max (Life Technologies, Carlsbad, CA, USA) at one day before nucleofection in six-well plates. An equimolar cocktail (total final concentration of 100 nM) of SASI_Hs01_0003-1409/ATAT1 and SASI_Hs02_0035-7514/ATAT1 (both purchased from Sigma) which target both isoform 1 and 3 of ATAT1 was transfected.

#### 4.3.2. Human Colon Cancer Cell Line

HCT116 cells were provided by RIKEN BRC and cultured in low-glucose D-MEM supplemented with 10% fetal bovine serum. For immunoblotting analysis, approximately 3.0 × 10^5^ cells/well were seeded onto six-well plates. For immunofluorescence studies, approximately 1.0 × 10^5^ cells/dish were plated on 3.5 cm glass-bottom dishes. In the experiments testing AOA, cells were plated one day before the experiments. For the knockdown of HDAC3, two siRNAs were used in an equimolar cocktail (at a total final concentration of 100 nM). For the first siRNA, the oligomers sense: ggaaugcguugaauauguc and antisense: gacauauucaacgcauucc (as previously described [80]) were annealed. The second siRNA, siHDAC3, was commercially obtained from Sigma (SASI_Hs01_00136351). The knockdown of FOXO3a (final concentration, 100 nM) was conducted using previously described siRNAs [8]. We used RNAi Max for the knockdown of HDAC3 and FOXO3a.

#### 4.3.3. Human Colon Epithelial Cells (HCECs)

Primary cultured normal human colonic epithelial cells (HCoEpiC) were purchased from ScienceCell Research Laboratories (Carlsbad, CA, USA). These cells were generated from human colonic tissues and cryopreserved at passage one. Experiments were performed within five passages. Cells were cultured in Colonic Epithelial Cell Medium (CoEpiCM; ScienceCell Research Laboratories) at 37 °C and 5% CO_2_ in accordance with the manufacturer’s instructions. For immunoblotting analysis, approximately 2.5 × 10^5^ cells/well were seeded onto six-well plates. For immunofluorescence studies, approximately 1.5 × 10^5^ cells/dish were plated onto collagen-coated 3.5 cm glass-bottom dishes. Transfection of HCECs with siRNAs was performed using TransIT-X2 Dynamic Delivery System (Mirus, Madison, WI, USA). For the knockdown of KL1, the Dharmacon siRNA smart pool (GE healthcare, Pittsburgh, PA, USA) was used, as described previously [18,19,81]. For the knockdown of ATAT1, the siRNA cocktail mentioned above was used. For the knockdown of p53, a previously described siRNA [19] was used. The final concentrations of the siRNAs were 100 nM for KL1, ATAT1, and p53. A fluorescent dye conjugated siRNA transfection showed nearly a 100% transfection efficiency.

Cell viability was determined following all treatments to rule out cell death using a Trypan Blue (Fujifilm Wako Pure Chemical Corporation) exclusion assay, as described previously [32].

### 4.4. Western Blotting and Immunoblotting of Colon Cancer Tumor/Normal Tissue Lysate Test Strip Arrays

Western blotting and subsequent quantification of the signals was performed using LumiCube (LIPONICS, Tokyo, Japan) as described previously [18,19]. Whole cell lysates were analyzed as described previously for the quantification of acetylated-tubulin [16,82]. For knockdown experiments, cells were transfected with siRNAs one day before ketone body treatments. All drug treatments were performed simultaneously unless otherwise mentioned. The protein amounts were quantified by densitometry using NIH ImageJ software. More than three independent experiments were performed for each set of analysis with quantification. Data represent the mean ± SD. Statistical analyses were done using the Student’s *t*-test.

Human colon tissue lysate test strips (ST7-6X-1 (male) and -2 (female)) were purchased from Protein Biotechnologies (Ramona, CA, USA). The strips were immunoblotted with anti-acetylated-tubulin and anti-α-tubulin antibodies in accordance with the manufacturer’s instructions using ECL reagents. All cases on the strip were stage I-IV adenocarcinomas. The following cases could be evaluated without experimental artifacts: T7-013, T7-017, T7-018, and T7-022 on the ST7-6X-1 strip and T7-037, T7-041, T7-044, and T7-046 on the ST7-6X-2 strip. Three independent experiments were performed for each blot. The expression levels were evaluated by densitometry with cut off values of 1.5 and 0.67. Data analysis was performed as done for the western blots.

### 4.5. Semiquantitative RT-PCR

Approximately 4.0 × 10^5^ HCT116 cells/well were seeded onto six-well plates and were treated with ketone bodies and cultured for two days. One microgram of total cellular RNA, extracted from cells by applying TRI Reagent (Molecular Research Center, Inc., Cincinnati, OH, USA), was transcribed into cDNA using All-In-One 5X RT MasterMix (Applied Biological Materials Inc., Richmond, BC, Canada). For the comparison of the ATAT1 mRNA levels, 1 μL of reaction product was analyzed. PCR was performed using EconoTaq Plus Green 2X Master Mix (Lucigen, Middleton, WI, USA) with the following conditions: 2 min at 94 °C, 30 cycles of 20 s at 94 °C, 20 s at 55 °C, 30 s at 72 °C, and a final incubation for 10 min at 72 °C, using an ASTEC PC320 thermocycler (Astec, Fukuoka, Japan). β-actin was used as an internal control (5′-primer: AACACCCCAGCCATGTACG, 3′-primer: CGCTCAGGAGGAGCAATGA). The MEC-17 (f) and MEC-17 (r) primers were used for ATAT1 amplification, as described previously [26]. Three independent experiments were performed. Statistical analyses were done using the Student’s *t*-test.

### 4.6. Immunofluorescence Techniques

Immunostaining experiments were performed as described previously [18,19]. For centromere staining with ACA, HCECs were fixed with ice cold methanol + acetone (1:1) for 15 min. Fluorescence signals were then detected using an Eclipse TE200 fluorescence microscope (Nikon, Tokyo, Japan) with Plan-Fluor 100 × (oil) and LU Plan-Fluor 20 × objective lenses with 1.3 and 0.45 apertures, respectively, and equipped with a CCD camera (Orca ER; C4742-95-12ERG; Hamamatsu, Shizuoka, Japan). The original magnifications were x1000 and x200, respectively. All images were captured using HCimageLive software (Hamamatsu). To quantify the microtubule levels, cells were simultaneously fixed and extracted to remove free tubulin and then immunostained as described previously [18,19]. Images to be compared were taken at identical settings of exposure time, brightness and contrast and analyzed with ZEN 2012 (blue edition) software (Carl Zeiss, Oberkochen, Germany). In the microtubule sensitivity test, we chose highly exogenous protein-expressing cells for the analysis. Measurements of the microtubule levels were taken as the total fluorescence intensity per cell using the intensity mean value analytical command in the ZEN software. Values were expressed as arbitrary fluorescent units (AFUs). Three independent experiments were performed for each set of analyses. Data represent the mean ± SD. Statistical analyses were done using the Student’s *t*-test.

### 4.7. ROS Detection

DCFDA was used as a cell-permeable fluorogenic probe to quantify the ROS level. This compound is rapidly de-esterified in cells and is oxidized to form fluorescent 2′,7′-dichlorofluorescein, which displays excitation/emission spectra of 492/515 nm. HCT116 cells were plated at about 1.0 × 10^5^ cells/dish onto 3.5 cm glass-bottom dishes, treated with ketone bodies, and cultured for two days. Cells were further incubated with 10 μM DCFDA for 30 min at 37 °C, rinsed twice with Hank’s balanced salt solution (HBSS) (Life Technologies) then maintained in HBSS and analyzed at a 200× magnification under a fluorescent microscope. ROS signals were then quantified as described previously [83]. The optimal microscope and camera settings were determined and left constant throughout the entire experiment. For each experimental condition, images from 10 random fields of 25–100 cells per field were collected in the phase contrast and fluorescent channels. The fluorescence signals were analyzed in the same manner as the images taken in the immunofluorescence studies. Three independent experiments were performed in each case. Data represent the mean ± SD. Statistical analyses were done using the Student’s *t*-test.

## Data Availability

Not applicable.

## Supplementary Materials

The following are available online at https://www.mdpi.com/article/10.3390/ijms22179397/s1.
