# Supplementary material for: The Aneugenicity of Ketone Bodies in Colon Epithelial Cells Is Mediated by Microtubule Hyperacetylation and Is Blocked by Resveratrol
Source: Int J Mol Sci. 2021 Aug 30;22(17):9397. doi: 10.3390/ijms22179397 (PMC8430621; doi:10.3390/ijms22179397)
Supplement: Supplementary file 1 [file ijms-22-09397-s001.zip › ijms-1290875-supplementary.pdf]

The aneugenicity of ketone bodies in colon epithelial cells is mediated by microtubule hyperacetylation and is blocked by resveratrol

Haruka Sudo and Akira Kubo

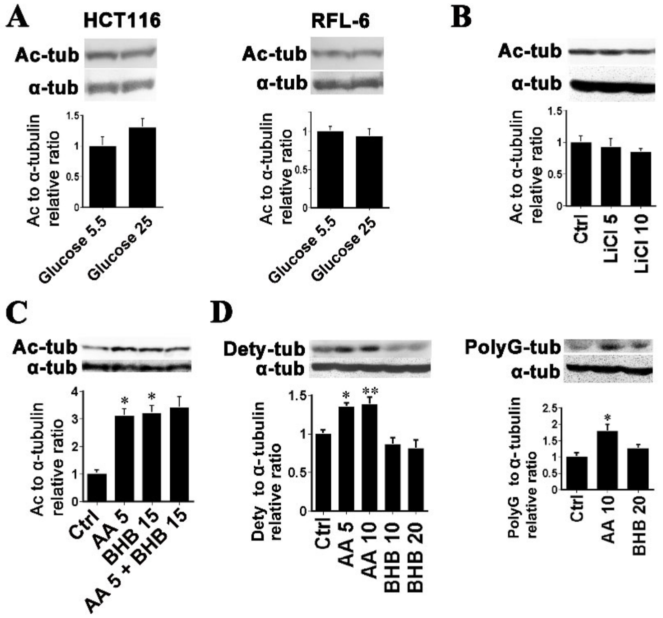

Figure S1. Additional results for ketone body-induced microtubule hyperacetylation. (A) HCT116 and RFL-6 cells were treated for three days with a final concentration of 25 mM glucose (Glucose) (comprising 5.5 mM originally present in the low-glucose D-MEM supplemented with an additional 19.5 mM) and whole cell lysates were then subjected to immunoblotting with the indicated primary antibodies. The relative Ac and α-tubulin ratios to the controls (exposed to the 5.5 mM glucose in the culture medium only with none added) were assessed. The relative ratios of acetylated-tubulin/α-tubulin to the controls were 1.3 ± 0.2, and 0.9 ± 0.1, for HCT116, and, RFL-6 cells, respectively. No significant change was found in either cell type. (B) HCT116 cells were treated with 5 or 10 mM of lithium chloride (LiCl) for three days, and whole cell lysates were then subjected to immunoblotting. No significant change was detected. (C) HCT116 cells were treated for three days with 5 mM AA, 15 mM BHB, or a combination of these agents (5 mM AA + 15 mM BHB) and

then subjected to immunoblotting. The relative ratios of acetylated-tubulin/α-tubulin to the controls were 3.1 ± 0.3, 3.2 ± 0.3, and 3.4 ± 0.4, for AA at 5 mM, BHB at 15 mM, and 5 mM AA + 15 mM BHB, respectively. No significant increase was observed upon exposure to the combined treatment compared with the AA or BHB treatments alone. (D) HCT116 cells were treated for three days with 5 or 10 mM AA, and 10 or 20 mM BHB, and whole cell lysates were then subjected to immunoblotting with the indicated primary antibodies (anti-detyrosinated-tubulin: Dety-tub and anti-polyglutamylated-tubulin: PolyG-tub). The relative modified tubulin to α-tubulin ratios to the controls are shown in the graphs below the images. AA treatments significantly increased both detyrosination and polyglutamylation while BHB treatments did not. The asterisks and double asterisk denote significant differences compared with the controls (Student's *t*-test, $P < 0.01$ and $P < 0.05$, respectively).

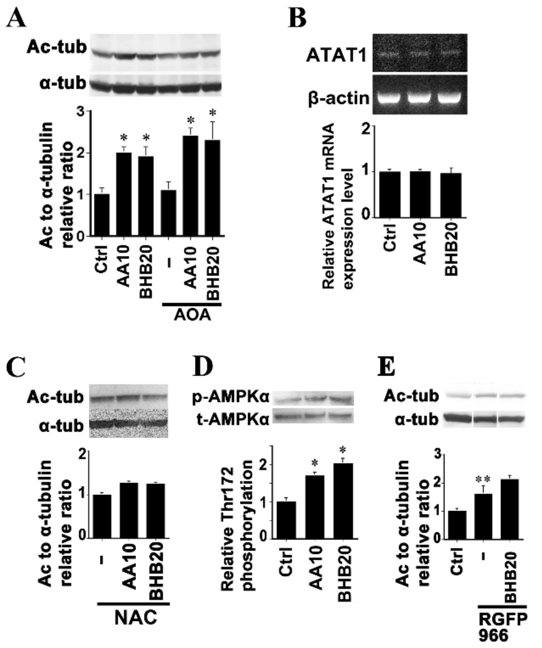

Figure S2. Mechanisms of ketone body-induced microtubule hyperacetylation. (A) In the presence of the TCA cycle entry inhibitor AOA (1 mM), HCT116 cells were treated with the indicated concentrations of AA and BHB and cultured for two hours. Subsequent western blot analysis revealed that either AA- or BHB-induced tubulin hyperacetylation was detectable even in this early period. No inhibitory effects of AOA on the hyperacetylation were evident for either of the ketone bodies. (B) Semiquantitative RT-PCR analysis of

ATAT1 mRNA expression upon stimulation with 10 mM AA or 20 mM BHB for two days. There was no significant difference in the relative mRNA expression levels. β-actin was used as a loading control. (C) In the presence of 2 mM NAC, HCT116 cells were treated for three days with 10 mM AA and 20 mM BHB, and whole cell lysates were subjected to immunoblotting. The relative Ac to α-tubulin ratios to the controls are indicated in the graph. There was no significant increase detected. (D) AMPK phosphorylation was analyzed in the total lysates of HCT116 cells that had been treated for three days with ketone bodies. Anti-phospho-AMPKα1/2 (Thr 172 phosphorylation) (p-AMPK) and anti-AMPKα1/2 (t-AMPK) were used. The latter antibody was used to detect of the total amount of direct substrate in this phosphorylation event. AA and BHB treatments significantly increased the relative phospho-AMPK/total-AMPK ratios compared with the controls to 1.7 ± 0.1 and 2.0 ± 0.1 for AA and BHB treatments, respectively. (E) In the presence of 10 μM RGFP966, a specific HDAC3 inhibitor, HCT116 cells were treated for three days with 20 mM BHB and analyzed by immunoblotting. A significant increase in tubulin acetylation was observed in the RGFP966-treated cells compared with the controls (double asterisk in the graph). Under exposure to this inhibitor, no significant increase was detected following BHB treatments. The asterisks and the double asterisk indicate significant differences compared with the controls (Student's *t*-test, *P*< 0.01 and *P*< 0.05, respectively).

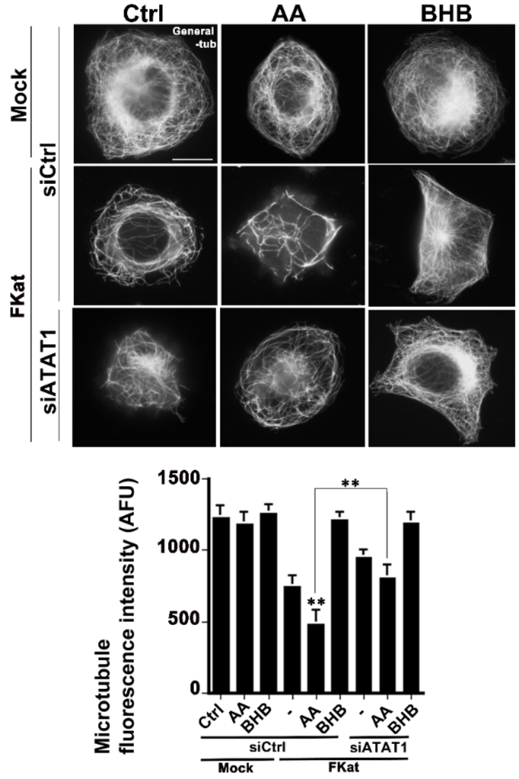

Figure S3. Converse effects of the AA and BHB ketone bodies on katanin-mediated microtubule severing in IMR90-SV cells. Representative images of microtubule sensitivity test results in IMR90-SV cells. Flag-katanin (FKat)-overexpressing cells showed significant microtubule reduction compared with the control mock-transfected cells (Mock). There was no difference in the microtubule reduction following a further control siRNA transfection. The AA treatment enhanced the microtubule reduction significantly (AA+FKat+siCtrl) compared with the untreated-FKat expressing cells. On the other hand, BHB treatment caused an inhibition in the FKat-induced microtubule reduction (BHB+FKat+siCtrl). In siATAT1-transfected cells, the enhancement seen in AA-treated cells was significantly inhibited (AA+FKat+siATAT1). The graph indicates the quantification data. AFU, arbitrary fluorescence unit. Scale bar, 10 μm. The double asterisks indicate significant differences (Student's *t*-test, $P< 0.05$).

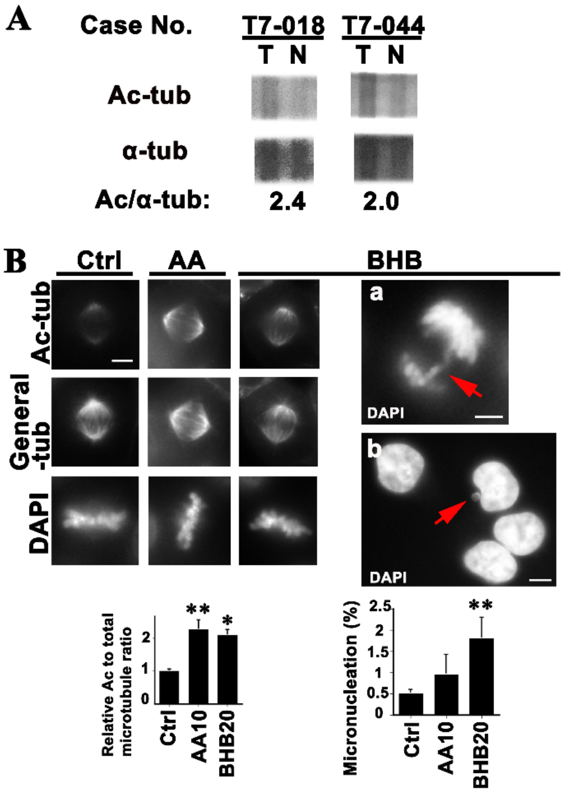

Figure S4. Tubulin acetylation status of human colon cancers and the micronucleation induced by ketone bodies in HCT116 cells via spindle tubulin hyperacetylation. (A) Human colon cancer tumor (T)/normal

(N) tissue lysate test strip arrays were immunoblotted with anti-acetylated-tubulin (Ac-tub) and anti-α-tubulin (α-tub). Ac/α-tub ratios (the Ac-tub/α-tub ratio in the tumor was divided by that of the neighboring normal tissue) are indicated below the images. Cases with the ratio of more than 1.5 are shown. Among 8 evaluable cases, 2 cases showed an increase (25%) while no others showed a less than 0.67 reduction. (B) Mitotic spindle hyperacetylation was induced by both 10 mM AA and 20 mM BHB. HCT116 cells were treated with ketone bodies for 3 days and subjected to immunofluorescence analysis with the indicated probes. Compared with the control mitotic spindles, both the AA- and the BHB-treated mitotic spindles showed a significantly higher level of microtubule acetylation, which was confirmed by the quantification results (graph). In the BHB-treated cells, the number of mitotic spindles with aberrant morphologies increased significantly compared with the control (BHBa; the red arrow highlights a chromosome bridge during anaphase). In agreement with the abnormal mitosis that was observed in the BHB-treated cells, a significant increase in micronucleation was also evident (BHBb; the red arrow indicates the micronucleus) (graph). The AA-treated cells also showed tendencies towards an increase in abnormal mitosis and micronucleation. Scale bars, 5 μm. The asterisk and double asterisks indicate significant differences compared with the controls (Student's *t*-test, $P< 0.01$ and $P< 0.05$, respectively).
